# Optimizing Magnet Spacing to Enhance Power and Energy Density in Magnetically Levitated Electromagnetic Vibration Energy Harvesters

**DOI:** 10.3390/mi16121404

**Published:** 2025-12-13

**Authors:** Madina Alimova, Elvira Kadylbekkyzy, Nurtay Albanbay, Aigerim Issimova, Rinat Ilesibekov, Bekbolat Medetov

**Affiliations:** 1 Department of Telecommunication Engineering, Almaty University of Power Engineering and Telecommunications, Baitursynuly Street 126/1, Almaty 050013, Kazakhstan; m.alimova@aues.kz (M.A.); e.kadylbekkyzy@aues.kz (E.K.); 2 Institute of Automation and Information Technologies, Satbayev University, Satbayev 22, Almaty 050013, Kazakhstan; r.ilesibekov@stud.satbayev.university; 3 Faculty of Physics and Technology, Department of Electronics and Astrophysics, Al-Farabi Kazakh National University, 71 Al-Farabi Ave., Almaty 050040, Kazakhstan; 4 Faculty of Physics and Engineering, Department of Radio Engineering, Electronics and Telecommunications, Gumilyov Eurasian National University, Satpayev 2, Astana 010008, Kazakhstan

**Keywords:** electromagnetic vibration transducer, magnetic levitation, electromagnetic generator, nonlinear dynamics, self-induction, bandwidth, energy density, IoT sensor nodes

## Abstract

In this study, we investigate a magnetically levitated electromagnetic vibration energy harvester (EMEH), in which a movable permanent magnet levitates between two fixed magnets with like poles facing the central magnet. We develop a nonlinear EMEH model and validate it experimentally, achieving strong agreement with the prototype (*R*^2^ = 0.95 for RMS EMF). Using this model, we perform a parametric analysis of excitation frequency and the spacing between the fixed magnets (*d*), yielding practical design criteria for geometry selection. The validated model predicts a narrow maximum; for the present configuration and parameter bounds, it occurs at *d* ≈ 28 mm with *P*_out_ ≈ 151.94 mW, and the corresponding energy density is *ρ_E_* ≈ 9.84 mW cm^−3^. These results yield a practical design rule for selecting *d* given target metrics and dimensional constraints, providing guidance for the design of compact, low-frequency harvesters powering autonomous sensor nodes.

## 1. Introduction

The authors of [1] discuss modern solutions for environmental monitoring based on LoRaWAN technology, which enables energy-efficient real-time data collection and analysis of environmental conditions. Such IoT systems demonstrate significant potential for creating autonomous sensor networks, where power can be supplied using energy harvesting technologies. Mechanical vibration energy harvesting technology enables the conversion of environmental vibrations into electricity and is considered a promising approach to the autonomous power supply of wireless sensors and IoT nodes [2,3,4,5,6].

One of the main reasons for the growing interest in vibration energy harvesters is the need to overcome the limitations of traditional batteries. Chemical power sources have a limited lifespan and require regular replacement or recharging, which complicates the long-term operation of distributed sensors without maintenance [7,8]. In contrast, vibration energy harvesters are able to extract an inexhaustible supply of ambient mechanical energy and convert it into electrical energy, providing a stable power supply for low-power devices. Various types of ambient energy (solar, thermal, wind, vibration, etc.) can be used for these purposes, but vibrations stand out for their widespread use in the technosphere [3,7]. Several physical principles are employed to convert vibrational energy into electrical energy, resulting in electrostatic [9], piezoelectric, and electromagnetic energy harvesters [10], among which electromagnetic vibration energy harvesters have garnered particular attention due to their comparatively high output power and superior efficiency at low operating frequencies [11].

In recent years, electromagnetic vibration energy harvesters (EMEHs) have been regarded as one of the most promising solutions for powering wireless monitoring systems, and their development has been the focus of extensive research efforts [12,13]. Conventional vibrational EMEHs are typically designed as linear resonant systems tuned to a fixed natural frequency. When the frequency of the external vibrations matches the resonant frequency, the generator’s relative motion amplitude reaches a maximum and, consequently, its output power is enhanced. However, even a slight deviation from the resonant frequency leads to a sharp decline in the energy conversion efficiency [14]. In practice, the spectrum of available ambient vibrations is often broad and shifted toward the low-frequency range, making precise resonance matching unlikely. As a result, narrowband resonant systems are unable to consistently generate sufficient power outside the narrow vicinity of their tuned frequency. For instance, it has been demonstrated that deviation of the excitation frequency from resonance causes a drastic (several orders-of-magnitude) reduction in the output power of a vibrational energy harvester [14].

Thus, the limited bandwidth of conventional linear EMEHs significantly hinders their applicability under real-world conditions, where the frequency content of ambient vibrations is distributed over a wide range and low-frequency components predominate [15]. To ensure a reliable power supply for wireless devices, it is necessary to broaden the operational frequency range of the energy harvester and maintain relatively stable output power across varying excitation frequencies. Several approaches have been proposed to extend the effective operating bandwidth of vibration energy harvesters. One direct method involves the use of multi-frequency (multi-degree-of-freedom) resonant systems. In such systems, multiple mechanical resonators with different natural frequencies (e.g., several spring-mass elements) are introduced, resulting in a combined response that exhibits multiple resonance peaks across the frequency spectrum. In the limiting case, a combination of several narrowband harvesters, each tuned to a different frequency, can collectively cover a broader frequency range [16]. An example of a linear approach to bandwidth expansion is presented in [17], where the authors developed an electromagnetic energy harvester featuring two closely spaced resonant frequencies. This approach enabled the combination of individual resonant responses to form a broader operational bandwidth without the use of nonlinear elements. However, it also increased system complexity and weight due to the inclusion of additional masses and elastic components.

The resonators in multi-degree-of-freedom systems are inevitably dynamically coupled, which may result in mutual interference and the emergence of power dips between the separated resonance peaks. In practice, it is often observed that the two resonances in a dual-mass system are spaced relatively far apart, resulting in a dip between them where the output power is low, making it challenging to achieve a truly uniform broadband response. As an alternative to the hardware-based duplication of resonators, frequency tuning of a single resonator—either actively or passively to match external conditions—has been proposed. Adaptive systems with tunable spring stiffness or tension —for example, by using magnetic fields, variable loading, or other adjustment mechanisms—have also been explored [18]. In [19], a magneto-spring structure is implemented to extend the effective operating frequency range of an energy harvester by adjusting the spring stiffness in accordance with different modes of human motion. This method enables tuning of the resonant frequency to match the current vibration frequency, thereby broadening the operational bandwidth. However, adaptive schemes increase structural complexity, and the tuning mechanism often consumes energy, which reduces the overall energy gain and system reliability. An alternative approach involves the use of nonlinear mechanics, which allows for bandwidth expansion through the system’s nonlinear response. In a linear resonator, output power drops sharply with frequency detuning, whereas nonlinear oscillators can maintain relatively high vibration amplitudes over a broader frequency range due to effects such as stiffness nonlinearity and bi- or multi-stability [20].

Another method of broadening the frequency range within the framework of nonlinear mechanics is based on the use of a two-degree-of-freedom nonlinear system. This approach is explored in [21]. The device’s design incorporates two fixed and two movable magnets. Owing to the presence of two degrees of freedom, the system exhibits both primary and secondary resonance frequencies, which enables an expanded operational frequency range and an increased number of output-power peaks. In [22], a nonlinear energy harvester with multiple degrees of freedom is investigated. This approach provides stable output power over a wide frequency range and is particularly effective under multi-frequency and chaotic vibrational excitations. Another method within the class of nonlinear bandwidth expansion techniques involves the use of nonlinear displacement constraints to limit the amplitude of motion.

The study presented in [23] examines the impact of wire diameter on the output performance of an electromagnetic energy harvester. The authors determined that an optimal diameter exists at which the device achieves a high electromotive force, reduced electrical damping, and efficient energy transfer to the load. These combined effects give rise to a nonlinear behavior known as the amplitude-limiting effect, which facilitates broadening of the harvester’s operational frequency range. Magnetic levitation-based vibration energy harvesters are of particular interest as illustrative cases of nonlinear systems. In such devices, the elastic suspension is implemented without a physical spring—instead, the repulsive force between permanent magnets is used, which is functionally equivalent to a magnetic spring effect. The absence of a rigid mechanical linkage offers several advantages: reduced friction and suspension losses, and an effectively variable magnetic stiffness that contributes to the widening of the operational frequency band. For instance, Hadas and Ondrusek [24] described a spring-less electromagnetic generator in which nonlinear stiffness is provided by a system of repelling magnets. It was demonstrated that this magneto-elastic configuration exhibits a broader bandwidth compared to its linear counterpart due to the “flattening” of the resonance peak [24]. Another approach involves the development of bistable or multistable oscillatory systems characterized by multiple potential wells. Transitions between stable equilibrium positions enable energy harvesting over a broader frequency range.

In a recent study, Abdelnaby and Arafa [14] presented a bistable electromagnetic energy harvester incorporating a pair of repelling magnets, which demonstrated a significantly wider effective excitation range compared to a linear generator, particularly under low-amplitude input vibrations. In another study [25], the authors propose a multistable structure in which the nonlinear characteristics of the system give rise to multiple resonance frequencies, each associated with distinct vibration modes. At low excitation levels, the device remains confined within a single potential well, while increased amplitude leads to inter-well oscillations. This frequency broadening strategy enables stable energy generation even under aperiodic and low-intensity excitations. Thus, nonlinear solutions are an effective means of expanding the operational bandwidth; however, this advantage is often accompanied by an increase in the system’s dynamic complexity. In nonlinear dynamical systems, noise and coupling effects can induce complex transient behaviors even when the deterministic attractor corresponds to a stable resting state. For example, recent studies on coupled FitzHugh–Nagumo oscillators have demonstrated the phenomenon of transient bursting, in which additive noise generates intermittent sequences of spikes followed by a return to rest [26,27]. Although the deterministic model converges to equilibrium, stochastic perturbations may cause trajectories to temporarily escape the local basin of attraction, resulting in bursting episodes with exponentially distributed lifetimes. Numerical simulations and experiments using analog electronic circuits confirmed that this noise-induced behavior is associated with the probabilistic nature of escape from the basin of attraction. Such findings provide a useful analogy for magnetically levitated energy harvesters, where nonlinear potential wells and magnetic repulsion forces can result in multistability or transient oscillatory regimes. Understanding these noise-sensitive transitions is crucial for improving the stability and energy conversion efficiency of electromagnetic harvesters operating near resonance or under irregular excitation. At the same time, strong nonlinearity may require relatively high excitation accelerations to overcome potential barriers (e.g., in bistable systems), which can limit the practical applicability of such designs.

A distinct class of approaches involves impact-based mechanisms and impulsive excitation. So-called impact energy harvesters utilize freely moving masses that periodically collide with stoppers or other masses during vibration. These impacts convert low-frequency ambient motion into high-frequency local oscillations of the generator—a phenomenon known as frequency up-conversion—which leads to increased output voltage and power. For example, Haroun et al. [28] demonstrated that a freely moving magnet impacting spring-loaded stoppers can generate up to ten times more power at a frequency of approximately 10 Hz than an equivalent linear resonator. Another approach was presented by Zhu et al. [15], who realized a multi-magnet electromagnetic generator for impulsive (step-like loading) base excitation. In their design, the central levitating magnet “jumps” between two limiting magnets. This multi-magnet configuration effectively achieves frequency up-conversion, transforming slow impulsive inputs into higher-frequency oscillations and enabling energy harvesting from step-induced vibrations [15]. In [29], the authors successfully extended the operational frequency range of the device up to 350 Hz, with a natural resonance frequency of around 75 Hz, by employing impact-based methods that enable excitation over a broad range of frequencies. The design features two oppositely oriented magnets that collide with the housing of the prototype during vibration. Despite the broadband response, the output power of the device remains relatively low. Multi-impact mechanisms and related designs have demonstrated the ability to broaden the energy-harvesting bandwidth and improve performance under low-frequency excitation. However, impact-based systems also have significant drawbacks: mechanical collisions lead to wear and reduced reliability, and a portion of the energy is inevitably lost during each impact. Furthermore, most impact-type vibration harvesters behave like linear resonators during non-collision periods, meaning that their output rapidly decreases when the excitation frequency deviates from resonance. Thus, although impulse-driven schemes can improve efficiency over specific frequency bands, sustaining high power output across a broad bandwidth remains challenging. Hybrid systems that combine different physical principles—such as piezoelectric–electromagnetic generators—have also been explored as a means of extending the operational range [30]. These systems offer certain advantages through the simultaneous use of multiple energy conversion mechanisms, but they require complex integration of heterogeneous components and impedance matching, which increases the overall design complexity.

A wide range of broadband vibration-harvesting concepts has been proposed in recent years, but many of them impose substantial practical limitations. Multi-frequency and multi-DOF architectures require additional masses and elastic elements, introducing dynamic coupling and increasing the likelihood of power dips between resonance peaks. Nonlinear bistable and multistable systems often require high excitation levels to trigger interwell transitions, which are not available in typical low-amplitude ambient vibrations. Impact-based mechanisms suffer from mechanical wear, energy dissipation during collisions, and long-term stability issues. These factors collectively restrict the suitability of most broadband solutions for compact, maintenance-free IoT devices, in which simplicity, reliability, and predictable behavior are essential.

A viable approach to adapting the resonant characteristics of a magnetically levitated electromagnetic energy harvester (MLEH) without increasing structural complexity is to modify the effective magnetic stiffness by adjusting the spacing between two fixed magnets. This spacing determines the nonlinear restoring force acting on the levitated magnet, thereby affecting both the resonance frequency and the resulting power–frequency response. Although the principle is conceptually straightforward, the quantitative relationship between this geometric parameter and the output characteristics of the harvester has not yet been systematically addressed in an analytical study. To bridge this methodological gap, we developed a dedicated analytical model that incorporates the system’s nonlinear dynamics and explicitly relates the spacing between the fixed magnets to key performance metrics, including resonance frequency and output power. The model accurately predicts the harvester’s behavior and provides a foundation for systematic optimization under given operational and geometric constraints. As such, it offers a practical and scalable design tool for compact, energy-autonomous devices operating in low-frequency vibration environments, particularly within the scope of Internet of Things (IoT) applications.

In this regard, this work focuses not on creating a new design but on conducting a detailed numerical and experimental analysis of the above-mentioned system with a view to determining the parameters at which the output power and energy density reach their maximum. The novelty of this research lies in identifying the optimal operating modes of a known device. A mathematical model of the system was developed, considering its nonlinear dynamics, and the model was verified based on laboratory measurements. Next, the dependence of the output power and energy density on the geometric parameters of the device (primarily the distance between the magnets, which determines the height of the system) was analyzed. Based on the data obtained, practical recommendations were formulated for the optimal design of energy harvesters of this type. Thus, this study fills an existing research gap and provides scientifically sound recommendations for the creation of more efficient vibrating electromagnetic energy harvesters with magnetic levitation.

The rest of this article is organized as follows: Section 2 presents the design of the device under study, which contains one levitating magnet between two fixed magnets of the same polarity. This section also describes the mathematical model, the numerical simulation method, the experimental setup, and the main parameters of the laboratory measurements. In Section 3, the numerical and experimental results are analyzed, the effect of the distance between the magnets on the output power and energy density is investigated, recommendations for the optimal configuration of the device are provided, and the main conclusions are presented. Section 4 includes the conclusions of the study, a discussion of its limitations, and directions for future research.

## 2. Materials and Methods

### 2.1. Device Concept and Design

**Figure 1:**
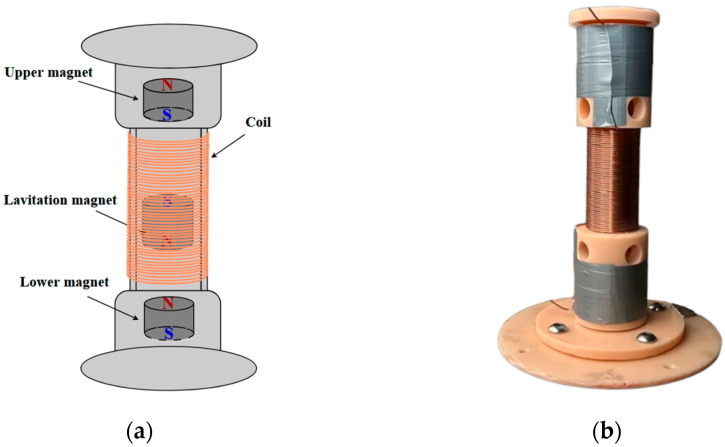
Structural diagram of the electromagnetic vibration energy harvester with one levitating magnet and two fixed magnets (upper and lower magnet) (**a**) and an image of the actual device (**b**).

The proposed electromagnetic vibration energy harvester includes two fixed magnets and one levitating permanent magnet. The fixed magnets are installed on the upper and lower bases and form a stable magnetic field. A levitating magnet is placed between them and held in magnetic levitation mode by the mutual repulsion of similarly oriented poles (Figure 1). This configuration has a few advantages: it does not require the use of traditional springs or suspensions, which eliminates friction and wear in the mounting nodes, and it provides a symmetrical “magnetic spring” for the levitating magnet with a relatively simple device design.

When external vibrations affect the base of the harvester, the levitating magnet starts moving and oscillates freely along the vertical axis around the equilibrium position. The repulsive forces from the upper and lower magnets act as an elastic restoring mechanism: when the magnetic mass is deflected upward or downward, a force arises that returns it to the center of equilibrium. In other words, magnetic repulsion can be modeled as the action of two springs symmetrically located relative to the equilibrium of the magnet.

To generate electrical energy, the device has an induction coil that covers the area of movement of the magnet. As the magnet oscillates, magnetic flux through the coil turns continuously. According to the law of electromagnetic induction, this induces an electromotive force (EMF) in the winding. When the electrical circuit is closed, the induced EMF creates a current flowing through the coil and the connected load. Thus, the mechanical energy of the oscillations is converted into electrical energy in the circuit.

**Figure 2:**
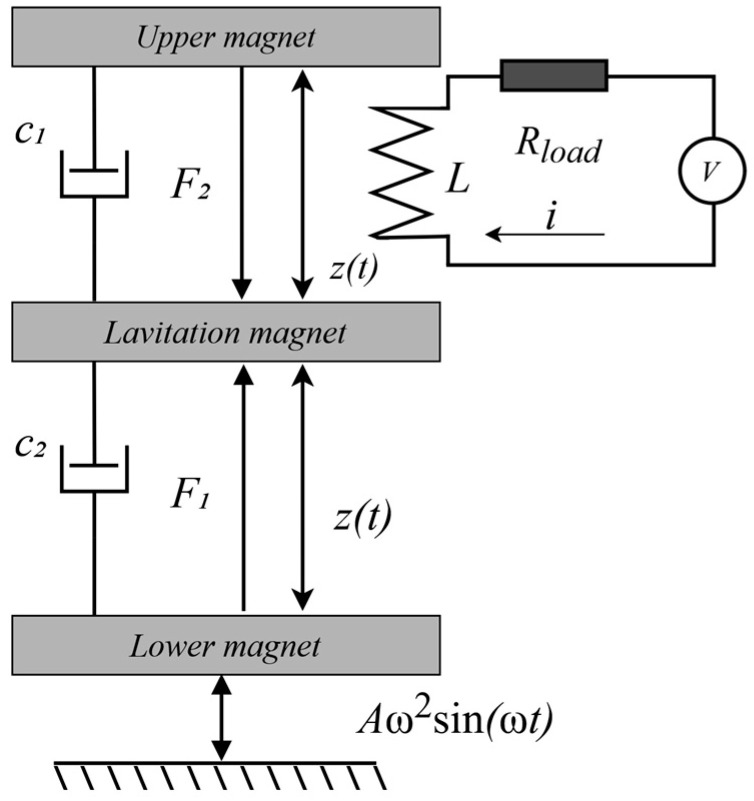
Electromechanical coupling model. Symbols: $F_{1}$ and $F_{2}$—magnetic forces from the upper and lower fixed magnets [N], respectively; $c_{1}$— and $c_{2}$ damping coefficients (mechanical + electromagnetic) [N·s/m], z(t)—displacement [mm]; L—coil inductance [H]; $R_{load}$—load resistance [Ω]; V—measured voltage [V]; $A\omega^{2}sin(\omega t)$—base acceleration [m·$s^{-2}$].

The current induced in the coil forms its own magnetic field (self-induction effect), which counteracts the change in magnetic flux caused by the movement of the magnet. In accordance with Lenz’s law, this results in an additional electromagnetic force acting on the levitating magnet, hindering its movement. An electromechanical feedback effect occurs: the oscillating magnet transfers part of its energy to the electrical subsystem, which manifests as electromagnetic damping of its movement (Figure 2). At high amplitudes and oscillation speeds, a stronger current is generated, and as a result, the counteracting force slowing down the magnet increases. Thus, the mechanical and electrical parts form a single closed system in which energy is continuously exchanged between the magnet and the electrical circuit as long as the vibration continues.

The concept of this device and the nature of the interaction between its subsystems served as the basis for subsequent numerical modeling. A complete verbal description of the energy harvester’s operating principle is necessary to correctly formulate the mathematical model and analyze the dynamic characteristics and energy efficiency of the system.

### 2.2. Mathematical Modeling

For a more detailed analysis of the device’s operation, a mathematical model was developed to describe its primary physical processes. This model predicts the behavior of the system according to various input parameters and evaluates the efficiency of converting vibrational energy into electrical energy.

A simple electromechanical diagram of the device is shown in Figure 2, which clearly illustrates its main components and operating principles. As the magnetic element moves inside the coil, an electromotive force (EMF) is generated in accordance with the law of electromagnetic induction. The coil terminal voltage is given by

(1) $$\varepsilon_{coil}\left(t\right)=\varepsilon_{ind}\left(t\right)-L\frac{di}{dt},$$

where $\varepsilon_{ind}(t)$ is the induced electromotive force (EMF) arising due to the change in magnetic flux passing through the coil; $\varepsilon_{coil}\left(t\right)$ is the voltage across the coil terminals; $L\frac{di}{dt}$ is the inductive voltage drop due to self-induction.

According to Faraday’s law, the induced EMF is defined as follows:

(2) $$\varepsilon_{ind}\left(t\right)=-\frac{d\Phi (t)}{dt}$$

where dΦ(t)/dt is the change in the magnetic flux passing through the loop over time. The magnetic flux through the loop is given by

(3) dΦ=S·dB,

where S is the cross-sectional area of a coil turn.

In our case, the change in B(t) is caused by the motion of the free magnet along the coil axis:

(4) $$\frac{dB(z)}{dt}=\frac{dB(z)}{dz}\cdot \frac{dz}{dt}=\frac{dB(z)}{dz}\cdot v(t),$$

where v(t)=dz/dt is the velocity of the magnet, and B(z) is the magnetic flux density along the axis of the coil.

To determine the change in the magnetic induction vector dB, it is necessary to know its dependence on the distance r between the magnet and the coil’s turns. The magnetic field along the axis of a cylindrical magnet is given by [31]

(5) $$B(z)=\frac{B_{r}}{2}\left(\frac{z+\frac{h_{m}}{2}}{{\left(z+\frac{h_{m}}{2}\right)}^{2}+r^{2}}-\frac{z-\frac{h_{m}}{2}}{{\left(z-\frac{h_{m}}{2}\right)}^{2}+r^{2}}\right)\;$$

where $h_{m}$ is the axial length (m) of the cylindrical magnet.

The derivative of magnetic induction along the *z*-axis is equal to

(6) $$\frac{dB(z)}{dz}=\frac{B_{r}}{2}\cdot \left(\frac{r^{2}}{{\left(r^{2}+{\left(z+\frac{h_{m}}{2}\right)}^{2}\right)}^{3/2}}-\frac{r^{2}}{{\left(r^{2}+{\left(z-\frac{h_{m}}{2}\right)}^{2}\right)}^{3/2}}\right)$$

**Figure 3:**
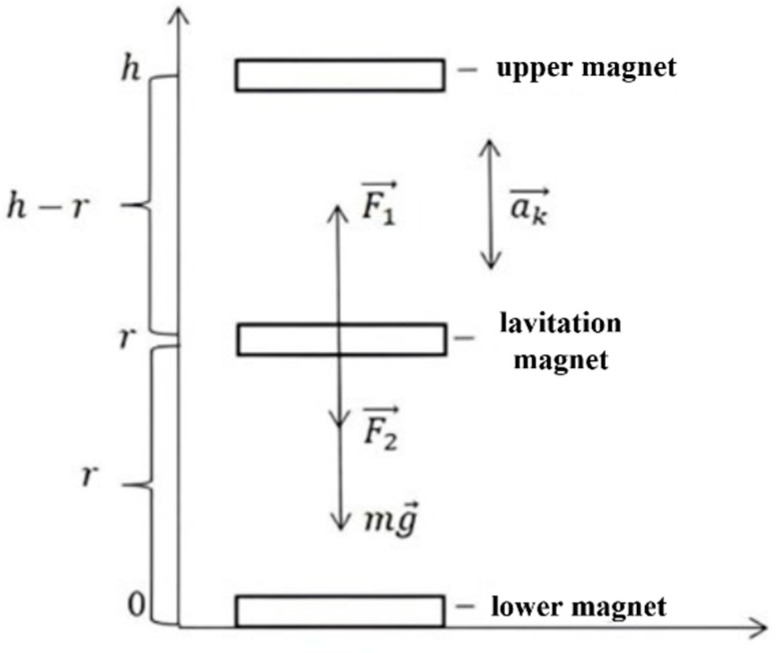
Forces acting on the moving magnet (*h*—coil height (distance between the lower and upper magnets)).

The total electromotive force (EMF) generated in the coil depends on the velocity and acceleration of the magnet’s motion relative to the coil. To determine these kinematic quantities, it is necessary to formulate equations describing the magnet’s motion. First, let us refer to Figure 3:

As shown in Figure 3, three forces act on the levitating magnet:

(1) Gravity $m\vec{g}$;
(2) The repulsive force of the lower magnet ${\vec{F}}_{1}$;
(3) The repulsive force of the upper magnet ${\vec{F}}_{2}$.

The forces ${\vec{F}}_{1}$ and ${\vec{F}}_{1}$ depend solely on the distance. The force between two coaxial cylindrical magnets is given by

(7) $$F(z)=\frac{\pi B_{r}^{2}r^{2}}{2\mu_{0}}\left(\frac{2(h_{m}+z)}{\sqrt{{\left(h_{m}+z\right)}^{2}+r^{2}}}-\frac{2h_{m}+z}{\sqrt{{\left(2h_{m}+z\right)}^{2}+r^{2}}}-\frac{z}{\sqrt{z^{2}+r^{2}}}\right)$$

The system is also subjected to an external excitation force caused by the harmonic oscillations of the device base, which are described by the following law:

(8) $$x=x_{0}sin(\omega t),\;\omega =2\pi f,$$

where $x_{0}$ is the amplitude and ω is the angular frequency.

Accordingly, the external inertial force acting on the magnet is defined as

(9) $$F_{shaker}(t)=-m\omega^{2}X_{0}cos(\omega t)$$

Considering all external and internal effects, the total force acting on the magnet is expressed as follows:

(10) $$F_{total}\left(t\right)=-F_{1}\left(z\right)+F_{2}\left(z\right)-mg+F_{shaker}\left(t\right)+F_{c}$$

where *Fc* is the damping force.

By substituting the corresponding expressions for the forces, we obtain a second-order differential equation describing the vertical motion of the magnet:

(11) $$m\frac{d^{2}z}{dt^{2}}=F_{1}-F_{2}-mg+mx_{0}\omega^{2}sin\left(\omega t\right)+F_{c}$$

By solving the second-order differential Equation (11), the position and velocity of the magnet can be determined. The variation in the position of the magnet leads to a change in the magnetic flux through the coil, which induces an electromotive force (EMF) according to Faraday’s law:

(12) $$\varepsilon_{i}(t)=-S\cdot \frac{dB(z)}{dz}\cdot v(t),$$

where S is the cross-sectional area of a coil turn, v(t)=dz/dt is the velocity of the magnet, and B(z) is the magnetic flux density along the axis of the coil.

The total EMF induced in a coil with N turns is determined by summing the contributions from all turns:

(13) $$\varepsilon_{i}(t)=-\sum_{i=1}^{N}S_{i}\cdot \frac{dB(z_{i})}{dz}\cdot v(t),$$

where $z_{i}$ is the position of the i-th turn of the coil.

Considering the phenomenon of self-induction, an additional electromotive force arises in the coil, which opposes the change in current. It is described by the following expression:

(14) $$\varepsilon_{s}=-L\frac{di}{dt},$$

where L is the inductance of the coil and i is the current flowing through the coil. The value of L is determined experimentally.

The rate of change of current can be determined from Ohm’s law as follows:

(15) $$i(t)=\frac{\varepsilon (t)}{R+R_{L}},$$

where ε(t) is the total EMF induced in the coil, $R_{L}$ is the coil’s internal resistance, and R is the resistance of the external load.

An approximate formula can be used to calculate the inductance of the coil:

(16) $$L=\frac{\mu_{0}\mu N^{2}S}{l},$$

where μ is the magnetic permeability, N is the number of turns of the coil, S is the cross-sectional area of the coil, and l is the length of the coil.

In this case, the motion-induced EMF is given by

(17) $$\varepsilon_{total}(t)=-\sum_{i=1}^{N}S_{i}\cdot \frac{dB(z_{i})}{dz}\cdot v(t)$$

The rate of change of current in Equation (14) is calculated using the following formula:

(18) $$L\frac{di}{dt}+(R+R_{L})i=\varepsilon_{total}(t)$$

Based on the above considerations and by arranging Equations (11), (17) and (18) into a unified framework, the resulting system of equations is as follows:

(19) $$\frac{dz}{dt}=v\;\frac{dv}{dt}=\frac{F_{1}-F_{2}-mg+F_{c}+mx_{0}\omega^{2}sin\left(\omega t\right)}{m}$$

Therefore, applying Newton’s second law to the motion of the levitating magnet enables the establishment of a correlation between its mechanical dynamics and the electromagnetic response of the system.

Thus, as a result of a step-by-step analysis of the physical interactions acting on the magnetic system, a complete mathematical expression for the resulting electromotive force was formulated, incorporating both inductive and self-inductive components. The resulting model accounts for the dependence of the EMF on the spatial distribution of the magnetic flux density, the velocity of the magnet, and the coil parameters.

Equation (19), together with the circuit Equation (18), governs the system dynamics and provides a basis for the numerical calculation of the output electrical characteristics.

#### 2.2.1. Evaluation Metrics

To quantitatively evaluate the accuracy of the model, the root mean square error (RMSE) metric was utilized, as defined by the following equation:

(20) $$RMSE=\sqrt{\frac{1}{N}\sum_{i=1}^{N}{\left(V_{model}(t_{i})-V_{exp}(t_{i})\right)}^{2}},$$

where N is the number of time samples, $V_{model}(t_{i})$ is the estimated value at the *i*-th sample, and $V_{exp}(t_{i})$ is the measured voltage value at the same time step.

In addition, the accuracy of the reproducing scalar characteristics was evaluated—specifically, the root mean square (RMS) value of the output voltage and the average power delivered to the load were calculated. The RMS voltage was calculated using the following formula:

(21) $$V_{RMS}=\sqrt{\frac{1}{N}\sum_{i=1}^{N}V_{i}^{2}},$$

and the average output power was determined as follows:

(22) $$P_{out}=\frac{V_{RMS}^{2}}{R_{load}}$$

The relative error in the average output power was calculated as follows:

(23) $$\delta_{P}=\frac{\left|{\bar{P}}_{model}-{\bar{P}}_{exp}\right|}{{\bar{P}}_{exp}}\times 100\%,$$

where $\bar{P}$ is the average power, defined as follows:

(24) $$\bar{P}=\frac{1}{T}\int_{0}^{T}\frac{V^{2}(t)}{R}dt$$

The model’s accuracy was evaluated by calculating the relative errors with respect to the following characteristic parameters:

(25) $$\varepsilon_{V_{RMS}}=\frac{\left|V_{RMS}^{model}-V_{RMS}^{exp}\right|}{V_{RMS}^{exp}}\cdot 100\%,$$

(26) $$\varepsilon_{P_{out}}=\frac{\left|P_{out}^{model}-P_{out}^{exp}\right|}{P_{out}^{exp}}\cdot 100\%$$

These metrics enabled an objective assessment of the degree of conformity between the numerical model and the actual performance of the device, as well as the identification of frequency ranges exhibiting the highest level of agreement.

Based on the simulation results, a pairwise comparison of output metrics was performed, as described below.

Average output power:

(27) $$P_{avg}=\frac{1}{T}\int_{0}^{T}\frac{{V(t)}^{2}}{R_{load}}dt\approx \frac{1}{N}\sum_{k=1}^{N}\frac{V_{k}^{2}}{R_{load}}$$

The output voltage amplitude (based on the peak-to-peak criterion):

(28) $$V_{pp}=maxV(t)-minV(t)$$

Resonant frequency:

(29) $$f_{res}=argmaxP(f)$$

Resonance peak bandwidth in terms of power (at the −3 dB level):

(30) $$∆f=f_{2}-f_{1},$$

where $P(f_{1,2})=\frac{1}{2}P_{max}$.

Based on the data obtained, the relative changes in the parameters due to the inclusion of self-induction were calculated and expressed as percentages:

(31) $$∆X=\frac{X_{with\;induction}-X_{without\;induction}}{X_{without\;induction}}\cdot 100\%$$

### 2.3. Numerical Simulation

#### 2.3.1. Methods and Implementation

Numerical simulation was performed based on the system of nonlinear differential equations formulated in Section 2.2, which describes the motion of a levitating magnet and electromagnetic induction processes. The solution was obtained using the fourth-order Runge–Kutta method, which was implemented in Python (version 3.12). An integration step of 10 µs was chosen based on a preliminary analysis of the convergence of the numerical method to ensure the required accuracy when calculating variables with high dynamic changes.

The following were calculated at each time step: the coordinate and velocity of the magnet, the induced and self-induced EMF in the coil, the current through the external load, and the instantaneous value of the output power. The average output power was determined by integration over time. The energy density of the device was calculated as the ratio of the average power to the active volume of the structure. All evaluation metrics were computed as defined in Section 2.2.1.

#### 2.3.2. Numerical–Experimental Validation

Numerical verification was carried out to evaluate the reliability of the developed mathematical model and to confirm its consistency with the actual operating conditions of the device. The primary objective of the verification phase was to compare the calculated results with experimental data obtained under identical external excitation conditions.

The base acceleration function *a(t)*, recorded during laboratory tests using a high-precision accelerometer mounted on a vibration platform, was employed as the input excitation in the numerical experiment. This function was directly imported into the numerical model and applied without modification, facilitating the exclusion of idealizations (e.g., sinusoidal excitation) and ensuring a realistic simulation of the physical process. Quantitative agreement metrics and representative comparisons between simulations and experiments are reported in Section 3.1.

#### 2.3.3. Analysis of the Contribution of Self-Induction (Method)

To quantify the contribution of self-induction, we performed a series of numerical experiments over the full excitation frequency range. In the model, the coil electromotive force (EMF) is represented as the sum of two terms: the motional EMF (due to relative motion of the magnet and coil) and the self-inductive EMF (due to current variation through the coil inductance). For each frequency point, we executed paired simulations with and without the self-induction term and, for each pair, computed the output voltage amplitude and the average load power using the same post-processing window and procedures as in Section 2.2.1. Quantitative comparisons following this protocol are reported in Section 3.2.

#### 2.3.4. Parametric Analysis of Fixed-Magnet Spacing and Excitation Frequency

To evaluate the influence of the fixed-magnet spacing *d* on the output characteristics of the device—namely, the amplitude of the induced electromotive force (EMF), the average output power *P_out_*, and the energy density $\rho_{E}$—a series of parametric numerical experiments was conducted. During the simulation, the value of *d* was systematically varied within a specified range, and computations were carried out for each configuration at different excitation frequency values f.

The structure of the parametric study is presented in Table 1, which shows the ranges of variation of the variables, the sampling step, and the number of points on each axis.

The following output parameters were evaluated in the numerical experiment: the amplitude of the induced electromotive force according to the peak-to-peak criterion (*Vpp*), the root mean square value of the output voltage (RMS), the average output power at equivalent load (*P_out_*), and the energy density of the device ($\rho_{E}$), defined as the ratio of the average output power to the active volume of the structure.

### 2.4. Experimental Setup

**Figure 4:**
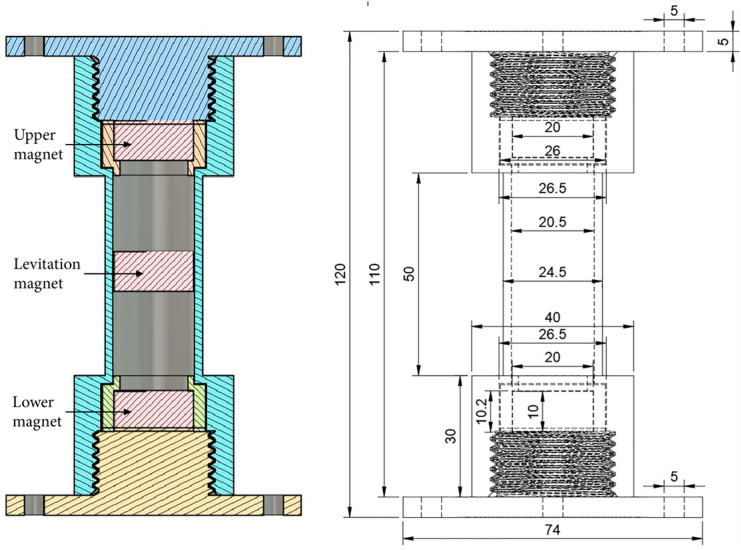
Structural diagram of the laboratory prototype of an electromagnetic vibrational energy harvester with a levitating magnet.

The harvester prototype (Figure 4) comprises a vertical guide tube made of a non-magnetic material, within which a cylindrical permanent magnet moves freely. An inductive coil is positioned in the central zone, encompassing the magnet’s trajectory.

The parameters of the magnet used are presented in Table 2. The magnet was fabricated from NdFeB alloy (Grade N42) and provided a residual magnetic induction of 1.11 T, sufficient to establish a stable repulsive magnetic field.

The device incorporates a single inductive coil. The coil’s geometric and electrical parameters are summarized in Table 3.

**Figure 5:**
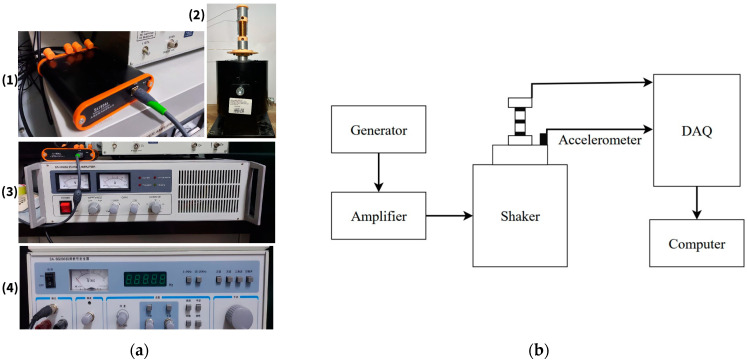
(**a**) Laboratory setup with a vibration shaker and a prototype of the harvester with a levitating magnet; (**b**) schematic diagram of the experimental setup.

Figure 5 shows the complete layout of the experimental setup, including a vibration shaker, a prototype device, measuring equipment, and control elements. The vibrations were generated by an SA-JZ020 (Figure 5a(2)) vibration shaker controlled by a signal generator SA-SG030 (Figure 5a(4)) and an SA-PA050 amplifier (Figure 5a(3)). The actual acceleration amplitude was monitored by an SAED0005B accelerometer mounted on the base of the setup. The signal was digitized and recorded by the SA1800 (Figure 5a(1)) data acquisition system. Before testing began, the accelerometer was calibrated with a load mass; the relative error in acceleration measurement did not exceed ±2%.

Experimental measurements were performed under stable external excitation; each operating point was recorded at least five times to ensure statistical reliability. The excitation parameters are listed in Table 4.

The signal applied to the shaker was simultaneously recorded by an accelerometer and used as input data in the numerical model, thereby eliminating the effect of excitation mismatch.

The experimental data obtained were subsequently employed to validate the numerical model and to determine the optimal geometric parameters of the device. A detailed comparative analysis is provided in Section 3.

## 3. Results and Discussion

### 3.1. Verification of the Mathematical Model

To verify the adequacy of the developed mathematical model of the electromagnetic vibration energy harvester, we performed a multifactor comparison of simulations against laboratory measurements, assessing both integral metrics (RMS voltage, output power, and resonance frequency) and instantaneous EMF time waveforms. The verification procedure is described in detail below.

The parameters subject to the greatest uncertainty, the electromechanical damping coefficient $c_{m}$ and the coil inductance, were calibrated at randomly selected experimental points. All remaining parameters were fixed from independent measurements and remained unchanged throughout the analysis.

**Figure 6:**
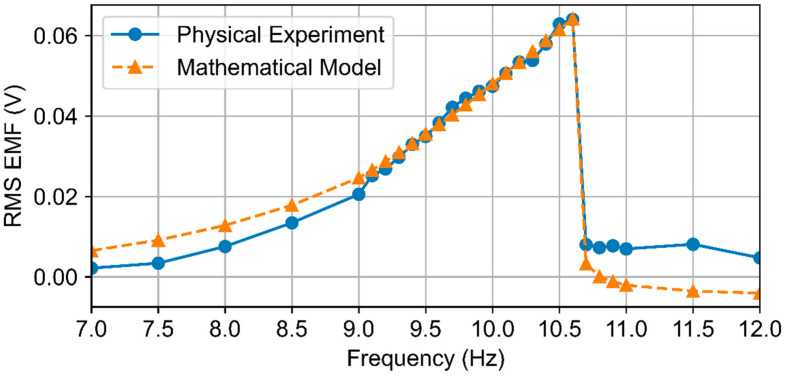
Verification of the model: calculated and measured EMF signals at resonance frequency.

Figure 6 shows the frequency response of the harvester—the dependence of the RMS EMF on the excitation frequency obtained experimentally and from the mathematical model. The simulation results closely follow the experimental data over the tested frequency range, accurately reproducing the resonance peak near 10.6 Hz and the overall amplitude–frequency shape.

Table 5 presents a comparison between the simulated and experimental RMS voltage and output power values at different excitation frequencies.

In the vicinity of the primary resonance (9.0–10.6 Hz), the model’s results match the measurements, with NRMSE *p* = 3.5% for RMS voltage and 3.1% for output power; the mean relative errors are 3.5% and 7.3%, respectively. At 10.7–10.8 Hz, the percentage errors are inflated by near-zero reference amplitudes and are not representative of the model’s fidelity. The waveform similarity is high (*R*^2^ = 0.95 for RMS_EMF(f)). Combined with the local time-domain agreement at resonance (Figure 6) and the frequency-domain comparison of RMS voltage and output power (Table 5), these results confirm the adequacy and validity of the proposed mathematical model of the electromagnetic energy harvester. The model can therefore be confidently used for further parametric studies, sensitivity analysis, and optimization of the device design.

### 3.2. Impact of Self-Induction on Output Characteristics

**Figure 7:**
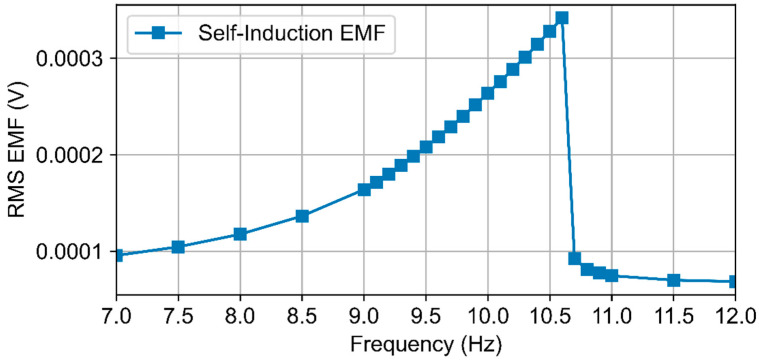
RMS value of the self-inductive EMF versus excitation frequency.

Using the developed model, we computed the self-inductive EMF contribution and evaluated its impact on the output characteristics (RMS voltage and output power). Figure 7 shows the calculated dependence of the self-inductive EMF (RMS) on the excitation frequency.

The curve exhibits a pronounced maximum in the resonance region (≈10.6 Hz), where the rate of change of the coil current is the largest.

**Figure 8:**
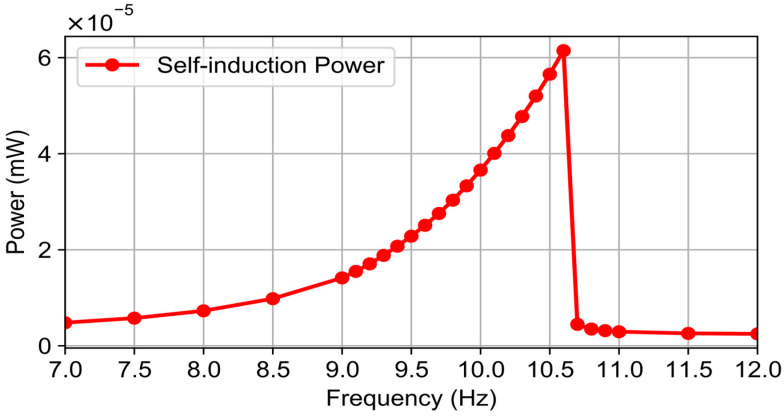
Power loss caused by self-induction as a function of excitation frequency.

To isolate the effect of self-induction, Figure 8 shows the power loss caused by self-induction plotted against the excitation frequency.

The curves show that including self-induction reduces the output power across the entire frequency range, with the most noticeable effect near resonance (≈10 Hz). In that region, the rate of current change is highest, so the self-inductive EMF (Lenz’s law) opposes the useful induced signal and decreases the amount of power delivered.

Table 6 summarizes the influence of self-induction on the harvester’s main output parameters (average load power and output-voltage amplitude). The results indicate that self-induction produces a measurable reduction in these quantities without shifting the resonance frequency or altering the −3 dB bandwidth.

Therefore, accounting for self-induction is necessary for reliable modeling and for selecting design parameters, especially when optimizing devices that operate near resonance under low-frequency vibrations.

### 3.3. Impact of Fixed-Magnet Spacing on Output Power

**Figure 9:**
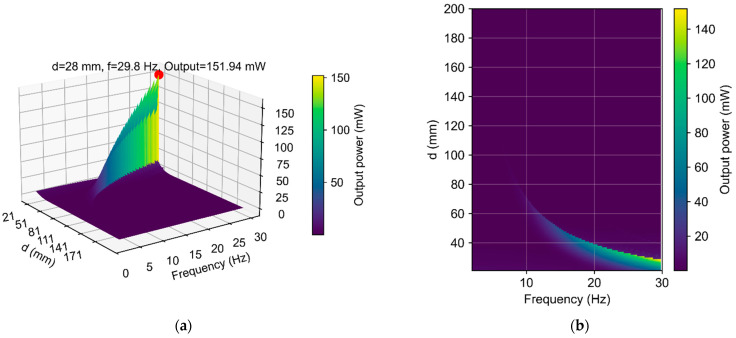
(**a**) Three-dimensional surface plot of output power ($P_{out}$) as a function of excitation frequency (*f*) and (**b**) fixed-magnet spacing (*d*).

Numerical modeling was performed to investigate how variations in the fixed-magnet spacing (d) affect the average output power ($P_{out}$) of the device. Figure 9 presents a 3D surface of the output power $P_{out}$ as a function of the excitation frequency *f* and fixed-magnet spacing d.

The dependence is non-monotonic: the surface contains several local peaks separated by low-power regions. The largest peak lies near the resonance band (around f≈29.8 Hz), while additional smaller peaks appear at neighboring d values. This behavior is expected: at a suitable d, the magnetic field coupling and the mechanical response align, resulting in higher induced voltage and load power. Without these conditions, detuning reduces $P_{out}$. For large d values, the power decreases gradually, consistent with a weaker field gradient in the coil region; for small d values, the motion range is limited, and power is also reduced. The peak region is narrow in f, indicating sensitivity to the excitation frequency; therefore, the selection of d should consider both the target frequency band and robustness to small frequency drifts. In summary, the surface provides a practical map: choose operating points in the dominant peak area for maximum $P_{out}$*,* and avoid flat regions where power is low.

### 3.4. Energy Density and Design Optimization

Energy density is a key performance metric for vibration energy harvesters, particularly under the spatial constraints typical of autonomous sensor systems. In contrast to absolute output power, it is normalized to the active volume of the device and therefore enables an objective comparison across designs of different dimensions.

**Figure 10:**
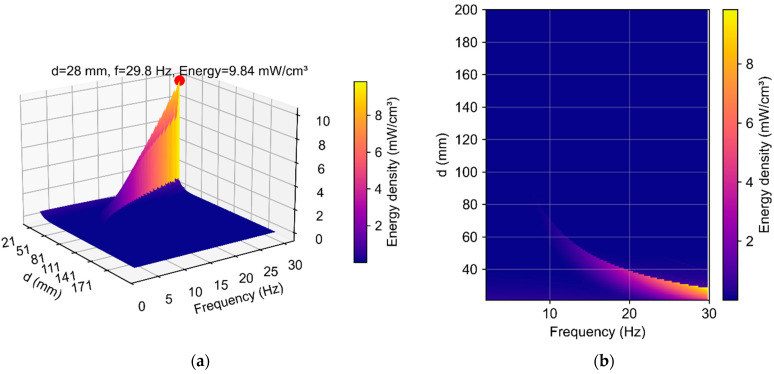
(**a**) Three-dimensional surface plot of energy density (*ρE*) as a function of excitation frequency (*f*) and (**b**) fixed-magnet spacing (*d*).

In this study, energy density was evaluated as the ratio of the average output power to the active volume, which increases proportionally with the fixed-magnet spacing (d). These tendencies are illustrated in the three-dimensional distribution shown in Figure 10.

The energy density *ρE* is mapped in Figure 10 as a function of the excitation frequency f and the fixed-magnet spacing d. The landscape exhibits a pronounced resonance ridge: for a given (d), *ρE* remains low at very low frequencies and rises toward a narrow peak near resonance, while increasing (d) shifts this ridge to lower frequencies and gradually reduces its amplitude. Physically, enlarging (d) weakens the net magnetic restoring profile, lowering the resonant frequency and limiting power, and increases the active volume approximately linearly; thus, volume normalization increasingly penalizes large spacings. The combined effect produces a single dominant maximum within the explored domain—near (d = 28 mm) and (*f* = 29.8 Hz) (highlighted in Figure 10a)—where operation close to resonance coincides with a still-moderate device volume. For smaller spacings (<25 mm), the resonance shifts to higher frequencies and the effective stroke diminishes, suppressing power. For larger spacings (>35–40 mm), the growing volume dominates, and energy density declines even if absolute power decreases more slowly. Hence, the optimal spacing is not set by power alone but by the balance between resonant amplification and volumetric normalization; in practice, Figure 10 serves as an “optimality map” for selecting d within a target frequency band, i.e., a narrow region in which high energy density is achieved with acceptable overall dimensions.

### 3.5. Comparison with Existing Solutions

In a comparative analysis, similar studies were considered. In [32], it was confirmed both theoretically and experimentally that combining two energy conversion mechanisms enhances overall efficiency through optimal damping distribution. However, the power density achieved was only 0.016 mW/cm^3^.

In another study [33], the authors combined a magnetic levitation electromagnetic harvester with a giant magnetoimpedance (GMI) sensor. The harvester used two fixed NdFeB magnets at the ends of a cylindrical frame and a levitating magnetic inertial mass moving inside a pickup coil (i.e., there were no mechanical springs). At its ≈10 Hz resonance and 0.5 g excitation, the device delivered up to ~1.4 mW into a 500 Ω load; given the reported active volume of ~12.7 cm^3^, this corresponds to a power density of ~0.11 mW/cm^3^. This configuration is compact and targets low-frequency vibrations (including human motion), and the co-integrated GMI sensor provides a linear, contactless readout of vibration amplitude. Under the present configuration and load, our model predicts an energy density of $\rho_{E}\approx 9.84$ mW/cm^3^ at d≈28 mm (Figure 10), which substantially exceeds the values reported in [32,33].

Compared with prior studies [32,33], this work contributes a single-parameter sizing rule for levitation-based electromagnetic harvesters: select the fixed-magnet spacing d by maximizing the energy density map $\rho_{E}\left(f,d\right)$ within the intended operating band. This principle yields a compact, scale-transferable prescription for designing high-specific-performance devices under tight volume constraints and directly informs subsequent configuration choices.

While the analytical model was experimentally verified in the 7–12 Hz frequency range, it also predicts output power across a much broader parameter space. The resonance peak near 29.8 Hz, identified as the global optimum, remains to be experimentally confirmed. Nonetheless, the strong agreement between model and experiment at lower frequencies supports the model’s reliability, and future work will focus on validating the high-frequency predictions.

## 4. Conclusions

This study examines how the spacing between the two fixed magnets (d) affects energy conversion efficiency in a magnetically levitated electromagnetic energy harvester (EMEH). A nonlinear EMEH model was developed and validated, showing strong agreement with experimental values ($R^{2}=0.95$). For the present configuration and parameter bounds, the model predicts a narrow peak at d≈28 mm with $P_{out}\approx 151.94$ mW and an energy density of *ρE* ≈ 9.84 mW·${cm}^{-3}$, yielding a practical design rule for selecting *d* to meet target performance and dimensional constraints.

The analysis was limited to a given frequency and amplitude range; the electrical load was considered fixed; and the dynamics were modeled as one-dimensional axial motion along the common magnet–coil axis, neglecting transverse and rotational degrees of freedom, backlash, and dry friction.

Future work should move beyond varying only d and jointly optimize d with coil parameters (inductance, resistance, and winding scheme) under dimensional constraints. It should also extend validation to the predicted global optimum in the peak-frequency region and assess performance under realistic excitation spectra (multi-frequency, random, and shock).

## Figures and Tables

**Table 1:** Structure of the experimental matrix.

Variable	Range	Step Size	Points
Fixed-magnet spacing *d*, mm	21–200	1	180
Excitation frequency *f*, Hz	2–29.9	0.1	280

**Table 2:** Parameters of the permanent magnet.

Parameter	Value	Unit
Magnet material	NdFeB (Grade N42)	-
Magnet shape	Cylinder	-
Magnet mass	42.0	g
Magnet diameter	19.5	mm
Magnet height	20	mm
Remanent flux density (Br)	1.11	T

**Table 3:** Geometrical and electrical parameters of the coil.

Parameter	Symbol	Value	Unit
Wire diameter	D	0.8	mm
Number of turns	N	55	turns
Inner diameter of the coil	Din	26.5	mm
Coil height	h	50	mm

**Table 4:** Experimental frequency ranges and measurement parameters.

Frequency Range	Step, Hz	Points
7–9 Hz	0.5	4
9–11 Hz	0.1	20
11–12 Hz	0.5	2

Note: frequency ranges are specified with a lower bound inclusive and an upper bound exclusive.

**Table 5:** Model verification—comparison of experimental and calculated values.

Frequency, Hz	RMS Voltage Model, V	RMS Voltage Experiment, V	Output Power Model, mW	Output Power Experiment, mW	Relative Error RMS, %	Relative Error Power, %
9.00	0.024623	0.020526	0.319113985	0.221737493	19.965	43.915
9.10	0.026550	0.025209	0.370995738	0.334467099	5.319	10.921
9.20	0.028694	0.026855	0.433340194	0.379575752	6.848	14.164
9.30	0.030929	0.029758	0.503481926	0.466060520	3.937	8.029
9.40	0.033212	0.032909	0.580547889	0.570007943	0.920	1.849
9.50	0.035515	0.034912	0.663866134	0.641482948	1.730	3.489
9.60	0.037853	0.038286	0.754116324	0.771479808	1.132	2.251
9.70	0.040242	0.042145	0.852324037	0.934849470	4.516	8.828
9.80	0.042755	0.044447	0.962100657	1.039741691	3.806	7.467
9.90	0.045364	0.046152	1.083092152	1.121052705	1.708	3.386
10.00	0.048018	0.047430	1.213555247	1.183999180	1.240	2.496
10.10	0.050680	0.050586	1.351836008	1.346796815	0.187	0.374
10.20	0.053340	0.053412	1.497430116	1.501507238	0.136	0.272
10.30	0.056019	0.053807	1.651664215	1.523801852	4.111	8.391
10.40	0.058720	0.057911	1.814729579	1.765067237	1.397	2.814
10.50	0.061434	0.062891	1.986383674	2.081756710	2.318	4.581
10.60	0.064179	0.064014	2.167839318	2.156732531	0.257	0.515

**Table 6:** Influence of self-induction on the output characteristics of the device.

Parameter	Without Self-Induction	With Self-Induction	Relative Error, %
Average Load Power, mW	1.089	1.079	0.9073
Amplitude of Output Voltage (Vp), V	0.06988	0.06954	0.4890

## Data Availability

Data will be made available upon request.

## Abbreviations

The following abbreviations are used in this manuscript:

EMEH	Electromagnetic Vibration Energy Harvester
IoT	Internet of Things
EMF	Electromotive Force
RMS	Root Mean Square
RMSE	Root Mean Square Error
LoRaWAN	Long Range Wide Area Network
